# A Dying Wife’s Dream

**Published:** 1891-10

**Authors:** 


### A DYING WIFE'S DREAM.

The mystery surrounding the sudden disappearance two years ago of William Short, who was at that time employed by the Long Island Railroad Company, as a car cleaner at Jamaica, L. I., has been solved. The discovery of his body in an abandoned well in the car yard was the result of a dream by his wife just before her death, last Tuesday.

At the time of Short's disappearance little was said in regard to the matter, Mrs. Short speaking of it only in her family and to relatives. Every night from the time of her husband's disappearance to the day of her death, Mrs. Short would place a light in the window and leave the door unlocked, believing that her husband would return. The support of the family, four boys and three girls, fell to the wife's lot and at times it was hard work for her to get along. Monday morning she attempted to leave the house, but sank into a chair exhausted and died there shortly after.

During the two years intervening between Short's disappearance and his wife's death, the children repeatedly declared that they believed the body of their father would be found at the bottom of the old railroad well. At the time of his disappearance Short's broom and pail were found near the uncovered well, and it is wondered at now that some examination of the well was not made.

Mrs. Short did not believe the statement of the children. Recently, however, she told some neighbors that she had been repeatedly awakened by a vision of her husband, who stood by her bedside and told her that he had fallen into the well. The vision appeared so real that several times she got up and lighted a lamp, after which the vision would disappear.

Mrs. Short's death revived these stories, and two men, John Magale and William Amberman, secured a fifty foot ladder, lowered it into the well and started to search the bottom. Magale made the first descent but soon reappeared with a frightened look on his face. When questioned as to the cause of his fright he replied that Short's body was at the bottom of the well.

The news of the finding of Short's body spread rapidly and a crowd soon gathered. A rope was procured, Magale made another descent, fastened the rope around the body, which was drawn up to the surface.

Notwithstanding the fact that it had been in the water for two years, it was in a very good state of preservation, no sign of decomposition being visible, and was recognized at once as that of the missing Short. This peculiar fact is attributed to the depth of the well and the large quantity of lime in the water.—*N. Y. Press*, Aug. 13.

